# Assessment of the Methodology That Is Used to Determine the Nutritional Sustainability of the Mediterranean Diet—A Scoping Review

**DOI:** 10.3389/fnut.2021.772133

**Published:** 2021-12-23

**Authors:** Carlos Portugal-Nunes, Fernando M. Nunes, Irene Fraga, Cristina Saraiva, Carla Gonçalves

**Affiliations:** ^1^CECAV-Veterinary and Animal Science Research Centre, Vila Real, Portugal; ^2^Food and Wine Chemistry Laboratory, CQ-VR-Chemistry Research Centre-Vila Real, University of Trás-os-Montes and Alto Douro, Vila Real, Portugal; ^3^Chemistry Department, School of Life Sciences and Environment, University of Trás-os-Montes and Alto Douro, Vila Real, Portugal; ^4^CITAB-Centre for the Research and Technology of Agro-Environmental and Biological Sciences, University of Trás-os-Montes and Alto Douro, Vila Real, Portugal; ^5^Department of Veterinary Sciences, School of Agrarian and Veterinary Sciences, University of Trás-os-Montes e Alto Douro, Vila Real, Portugal; ^6^CIAFEL—Research Center for Physical Activity, Health and Leisure, Faculty of Sports, University of Porto, Porto, Portugal; ^7^Biology and Environment Department, School of Life Sciences and Environment, University of Trás-os-Montes e Alto Douro, Vila Real, Portugal

**Keywords:** Mediterranean diet (MedDiet), nutritional sustainability, health indicator, environmental footprint (EF), diet impact

## Abstract

Mediterranean diet (MedDiet) is often used as an example of a sustainable diet that promotes a sustainable food system. MedDiet presents low environmental impacts, is characterized by high sociocultural food values, allows for positive local economic returns, and presents major health and nutrition benefits. Previous studies have not systematically examined the methodological assessment of MedDiet nutritional sustainability. In our study, we review the methodological assessment of nutritional sustainability, filling a crucial gap in the literature that can inform the state of the art regarding the cross-disciplinary assessment of MedDiet nutritional sustainability. Through a systematic search on PubMed and Scopus, we identified 28 studies, published between 2013 and 2021, that dealt with the MedDiet nutritional sustainability. Studies that assessed the sustainability of MedDiet based on dietary consumption data, studies that explored the MedDiet sustainability resorting to dietary scenarios, and studies with a mixed approach (dietary consumption and dietary scenarios) and proposals of methodological approaches to assess the MedDiet nutritional sustainability were summarized. We identified 24 studies exploring the dimensions of nutritional sustainability of the MedDiet, and 4 proposing the methodological approaches to assess the MedDiet nutritional sustainability or the sustainability of MedDiet typical agro-foods. From the 24 studies exploring the sustainability of MedDiet, none fully addressed the complexity of the four dimensions of nutritional sustainability (environmental, economic, socio-cultural, and health-nutrition). One of the methodological proposals to assess the MedDiet nutritional sustainability contemplated on the four dimensions of nutritional sustainability, as well as one of the methodological proposals to assess the sustainability of typical agro-foods of MedDiet. Environmental sustainability was the most well-studied dimension, while no study focuses on the socio-cultural dimension of sustainability. Our study reviewed for the first time the assessment of nutritional sustainability of MedDiet. To the best of our knowledge, no research has been made assessing MedDiet in all the dimensions of the complex concept, that is nutritional sustainability. Integrating health and nutrition, environmental, economic, and socio-cultural considerations across scales and contexts can offer a more complete understanding of the opportunities and barriers to achieving nutritional sustainability not only in MedDiet but also in other dietary patterns and food products.

## Introduction

Recently, the EAT-Lancet Commission identified food as the single strongest lever to optimize human health and environmental sustainability on Earth (1). Sustainable diets have emerged as a key issue in nutrition and public health (2). The notion of “sustainable diets” was proposed in 1986 by Gussow and Clancy to endorse diets that would be healthier for the environment as well as for consumers (3). Abandoned for several years, the interest in this concept has been gaining attention recently. In 2010, FAO in collaboration with Bioversity International reached a scientific position on the definition of sustainable diets: “Sustainable diets are those diets with low environmental impacts which contribute to food and nutrition security and to healthy life for present and future generations. Sustainable diets are protective and respectful of biodiversity and ecosystems, culturally acceptable, accessible, economically fair and affordable; nutritionally adequate, safe, and healthy; while optimizing natural and human resources” (4).

Sustainable diets are person-centered and are the last event in a chain that encompasses production, processing, distribution, and consumption of food and in their turn, define a food system. A high level panel of experts of the Committee on World Food Security defined a sustainable food system as “a food system that ensures food security and nutrition for all in such a way that the economic, social, and environmental bases to generate food security and nutrition of future generations are not compromised” (5). Sustainable diets and sustainable food systems are two closely interrelated notions. The contribution of the diet to the sustainability of the food system is what characterizes the sustainability of the diet, and sustainable diets are not only an objective but an essential means to achieve a sustainable food system (6).

Nutritional sustainability is defined as “the ability of a food system to provide sufficient energy and the amounts of essential nutrients required to maintain good health of the population without compromising the ability of future generations to meet their nutritional needs” (7, 8) and combines in one concept, aspects from sustainable diets and sustainable food systems. Nutritional sustainability is an interesting concept that not only sets environmental sustainability as a baseline level for balanced nutrition but also aims for the sustainability of the food system and calls for a more accurate assessment of the capacity of the environment for the development of more efficient nutrition solutions balanced within the limits of sustainability (8). Similar to the concept of sustainable food systems, nutritional sustainability also recognizes that ecological, social, and economic aspects must be balanced to support the sustainability of the overall food system but also acknowledged its contribution to health and nutrition present in the definition of sustainable diets (4–7).

Mediterranean diet (MedDiet) is often used as an example of a sustainable diet (9, 10) that promotes a sustainable food system (11, 12). MedDiet is a dietary pattern rich in cereals, fruits, vegetables, legumes, tree nuts, seeds, and olives, with olive oil as the principal source of added fat, along with high to moderate intakes of fish and seafood, moderate consumption of eggs, poultry and dairy products (cheese and yogurt), low consumption of red meat, and a moderate intake of alcohol (mainly wine during meals) (13, 14). MedDiet is the heritage of millennia of exchanges of people, cultures, and foods of all countries around the Mediterranean basin. It has been the basis of food habits during the 20th century in all countries of the region, based on Mediterranean agricultural and rural models (13). According to UNESCO, MedDiet involves a set of skills, that concerns, not only the sharing and consumption of food, but also knowledge, rituals, and traditions concerning crops, harvesting, fishing, animal husbandry, conservation, processing, and cooking. MedDiet is a way of life guided by respect for diversity, which emphasizes values of hospitality, neighborliness, intercultural dialogue, and creativity (15). Since its identification, MedDiet has been considered a healthy diet. Robust evidence suggests that adherence to the MedDiet is associated with a reduced risk of overall mortality, cardiovascular diseases (CVDs), coronary heart disease, myocardial infarction, overall cancer incidence, neurodegenerative diseases, and diabetes (16).

Based on its intrinsic characteristics, MedDiet presents several sustainability benefits. Considering the three dimensions of sustainability (environmental, social, and economic), MedDiet presents low environmental impacts, is characterized by high sociocultural food values, and allows for positive local economic returns. Furthermore, when talking about dietary patterns, a fourth dimension has been added, which is health and nutritional sustainability, which MedDiet also fulfills with major health and nutrition benefits (17).

Assessing the sustainability of the diets and/or nutritional sustainability is a challenging task. Despite the increased attention paid to nutritional sustainability and/or sustainable diets and the importance of clearly and comprehensively measured sustainability, it is not clear how the different components of sustainable diets and/or nutritional sustainability are prioritized or operationalized (18). The assessment of MedDiet sustainability has not been different. To the best of our knowledge, previous studies have not systematically examined the methodological assessment of MedDiet nutritional sustainability. Previous reviews have emphasized that studies have examined exclusively the environmental impacts of diets rather than assessing the many other components of sustainable diets (19, 20). Cross-disciplinary studies on environmental, economic, socio-cultural, and health-nutrition sustainability dimensions of the Mediterranean diet are a critical need (10).

The overall aim of this study is to provide a summary of the methodological assessment of nutritional sustainability in the context of MedDiet available in the scientific literature. More specifically, the objectives are to

(i) analyze the methodological differences in the assessment of nutritional sustainability
(ii) identify methods to combine nutrition indicators and sustainability indices, and to
(iii) explore the comprehensiveness of those indices to assess nutritional sustainability.

To our knowledge, this is the first scoping review focusing on the methodological assessment of MedDiet nutritional sustainability, filling a crucial gap in the literature that can inform the state of the art regarding the cross-disciplinary assessment of MedDiet nutritional sustainability.

## Methods

### Literature Search

The study design and analysis of this scoping review follow the Preferred Reporting Items for Systematic reviews and Meta-Analyses extension for Scoping Reviews (PRISMA-ScR) (21). The review protocol was not registered.

The search was made in Scopus and PubMed in October 2021 using the following search queries:

- Scopus: ;(title-abs-key (food) or title-abs-key (diet^*^) or title-abs-key (nutri^*^) and title-abs-key (sustain^*^) and title-abs-key (Mediterranean)];
- PubMed: ((((food[title/abstract]) or (diet^*^[title/abstract])) or (nutri^*^ [title/abstract])) and (sustain^*^[title/abstract])) and (Mediterranean [title/abstract]).

The search strategy was constructed based on the population, intervention, comparison, and outcome (PICO) framework. Table 1 provides a description of the PICO framework.

**Table 1:** Population, intervention, comparison, and outcome (PICO)framework.

Population or problem	Adults and youth aged 2 years and older
Intervention or Exposure	MedDiet
Comparison	Other dietary pattern or lower adherence to MedDiet
Outcome	Sustainability •Environmental indicators •Economic indicators •Socio-cultural indicators •Health-nutrition indicators

No time frame was set during the search to obtain a more comprehensive search of relevant published literature data. The literature search was limited to journal articles. Title, abstract, and keywords were searched in Scopus and, title and abstract were searched in PubMed. Articles reviewed were limited to English-language articles published in peer-reviewed scientific journals. Study protocols, gray literature, and conference abstracts were excluded. Articles included in this review were further limited to those using the following methodology:

(i) Assessment of MedDiet sustainability (alone or in comparison with other dietary patterns) using dietary consumption data;
(ii) Assessment of MedDiet sustainability (alone or in comparison with other dietary patterns) based on dietary scenarios;
(iii) Methodological proposals to assess the MedDiet nutritional sustainability of food, meals, or diets.

Determination of articles that met these inclusion criteria was made based on the information available in the titles and abstracts of the publications and in a later stage based on full text.

### Synthesis of Results

The assessment of reviewed articles was made from a research approach and methodological perspective. Studies that assessed the sustainability of MedDiet based on food consumption surveys data (3.2), studies that explored the sustainability of MedDiet resorting to dietary scenarios based on recommendations (3.3–dietary scenarios studies), studies with a mixed approach (3.4–food consumption surveys and dietary scenarios) and proposals of methodological approaches to assess MedDiet nutritional sustainability (3.5) were summarized.

All relevant information from eligible studies was collected using a data extraction sheet. For the studies that assessed the sustainability of MedDiet based on dietary consumption data, the following data were extracted: (i) study design (cross-sectional, longitudinal, or experimental), (ii) participant demographics (type of participants, sample size, and location), (iii) dietary patterns analyzed, (iv) sustainability indicators, and (v) findings. For the studies that resorted to dietary scenarios and for studies with a mixed approach, the study design was not relevant; therefore, it was not reported. Also, for the studies that resorted to dietary scenarios, participant demographics were not applicable; nevertheless, the location of the study was recorded. Given the type of works to be included in this review, no critical appraisal was performed.

## Results

### Study Selection and Characteristics

**Figure 1:**
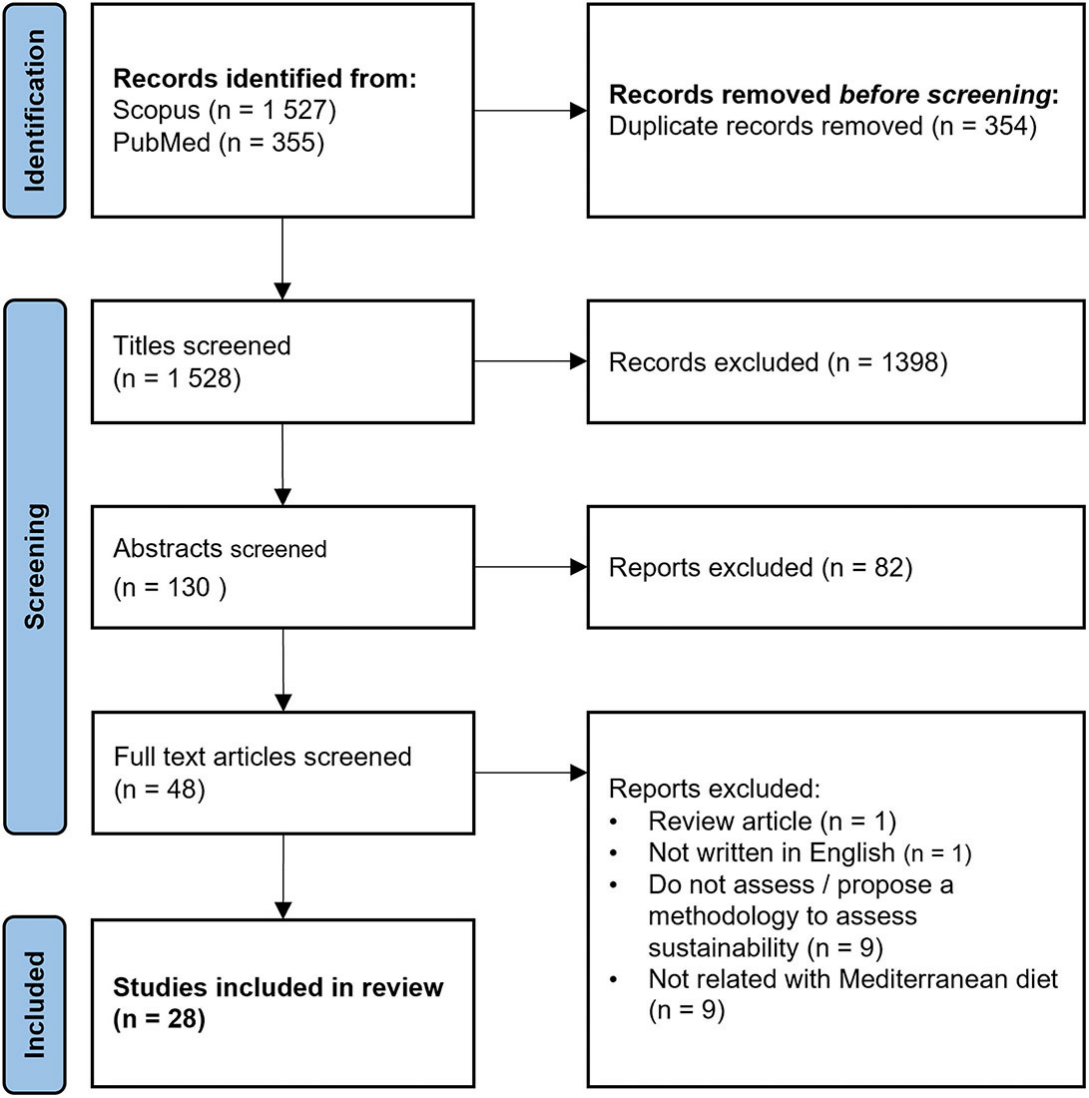
Literature search and selection of articles in the review.

The literature search identified 1,528 articles after duplicates removal. A total of 148 articles were excluded based on title and abstract screening. Full texts of the remaining 48 articles were examined in detail accounting for the inclusion and exclusion criteria. From those, 28 studies met the inclusion criteria. Details are outlined in the PRISMA flow diagram of the selection process (Figure 1).

The identified studies were published between 2012 and 2021. A total of 28 records met the eligibility criteria: 9 studies assessed the sustainability of MedDiet based on food consumption data (22–30), 11 studies assessed the MedDiet sustainability using dietary patterns recommendations (dietary scenarios) (31–41), 4 studies used a mixed approach (dietary consumption data vs. dietary scenarios) (42–45), and 4 studies proposed methodological approaches to assess sustainability within the MedDiet (46–49). All 28 articles reviewed were found to be transparent and provided the information required for our analysis.

### MedDiet Sustainability Based on Dietary Consumption Data

Out of the 28 articles included in this review, 9 analyzed MedDiet sustainability based on dietary consumption data. Relevant information from these articles is summarized in Table 2.

**Table 2:** Summary of studies reporting MedDiet sustainability using dietary consumption data.

**References**	**Study type**	**Participants; n**	**Location**	**Dietary patterns**	**Sustainability indicators**	**Findings**
Llanaj et al. (22)	Cross-sectional observational study	Young adults; n = 289	Albania	•MedDiet •DASH •EAT-Lancet reference diet	•Cost	•Better adherence to DASH, EAT-Lancet reference diet or MedDiet was not associated with dietary cost.
Grasso et al. (23)	Experimental (Clinical trial)	Adults; *n* = 744	Netherlands, United Kingdom, Germany and Spain	•Food-related behavioral activation therapy applying MedDiet guidelines (*n* = 373) •No intervention (*n* = 371)	•GHGs emissions •Land use •Energy use •pReCiPe score	•The intervention group reported increased intakes of vegetables, fruit, fish, pulses/legumes and whole grains, and decreased intake of sweets/extras relative to control group. •This effect on food intake resulted in no change in GHGs emissions, land use, and pReCiPe score, but a relative increase in fossil energy use. •A shift toward a healthier Mediterranean-style diet does not necessarily result in a diet with reduced environmental impact in a real-life setting.
Rosi et al. (24)	Longitudinal observational study	School children; *n* = 172	Italy	•MedDiet	•CF •EF	•CF and EF were higher during winter, and the main dietary contributors were red and processed meat for both indexes. •A small positive correlation was observed between adherence to the MD and total CF and EF.
Grosso et al. (25)	Cross-sectional observational study	Adults; *n* = 1,806	Italy	•MedDiet •DASH •Nordic diet •AHEI •DQI-I	•Land use •Water use •Energy use •GHGs emissions •Sustainability score	•Animal products (dairy, egg, meat, and fish) represented more than half of the impact on GHG emissions and energy requirements. Meat products were the stronger contributors to GHG emissions and water use. Dairy products were the stronger contributors to energy use. Cereals were the stronger contributors to land use. •All patterns investigated, except for DASH, were linearly associated with the sustainability score. •Higher adherence to MedDiet and AHEI was associated with lower GHGs emissions. •DQI-I was associated with lower land use. •Nordic diet was associated with lower land and water use.
Fresán et al. (26)	Longitudinal observational study	University graduates; *n* = 18,429	Spain	•MedDiet •Western dietary pattern •Provegetarian dietary pattern	•Rate advancement period (healthiness) •Cost •Environmental footprints index •Overall sustainable diet index	•The MedDiet exhibited the best rate advancement period (3.10 years gained for the highest *vs*. the lowest quartile), while the Western pattern was the unhealthiest pattern (1.33 years lost when comparing extreme quartiles). •Regarding EF index, Provegetarian pattern scored best when comparing extreme quartiles, whereas the Western pattern was the most detrimental pattern. •Regarding monetary costs, the Western pattern was the most affordable pattern (€5.87/day, for the upper quartile), while the MedDiet was the most expensive pattern (€7.52/day). •The MedDiet was the most overall sustainable option, closely followed by the Provegetarian pattern.
Naja et al. (27)	Cross-sectional observational study	Adults; *n* = 2,610	Lebanon	•MedDiet	•Water use •Energy use •GHGs emissions	•Two of the four MedDiet scores were associated with lower water use. •For GHGs emissions, significant inverse associations were observed with all MedDiet scores. •Energy use was not associated with MedDiet scores.
Naja et al. (28)	Cross-sectional observational study	Adults; *n* = 337	Lebanon	•Lebanese-MedDiet pattern •Western dietary pattern •High-Protein dietary pattern	•Water use •Energy use •GHGs emissions.	•The Lebanese-MedDiet had the lowest water use and GHGs emissions per 1,000 Kcal. •The highest energy use was that of the Western dietary pattern, followed by the Lebanese-MedDiet and the High-Protein dietary pattern.
Fresán et al. (29)	Longitudinal observational study	University graduates; *n* = 20,363	Spain	•MedDiet	•Land use •Water use •Energy use •GHGs emission •Sustainability score	•Better adherence to the MedDiet was associated with lower land use, water consumption, energy consumption and GHGs emission.
Seconda et al. (30)	Cross-sectional observational study	Adults; *n* = 22,866	France	•Conventional consumers and non-MedDiet followers (Conv–NoMedDiet; *n* = 14,266) •Conventional consumers and MedDiet followers (Conv–MedDiet; n = 3,498) •Organic consumers and non-MedDiet followers (Org–NoMedDiet; *n* = 2,532) •Organic consumers and MedDiet followers (Org–MedDiet; *n* = 2,570)	•PANDiet •mPNNS-GS •Dietary diversity score •Plant/animal protein intake ratio •Cost	•The adherence to nutritional recommendations was higher among the Org–MedDiet and Conv–MedDiet groups compared to the Conv–NoMedDiet group (using the mPNNS-GS). •The mean plant/animal protein intake ratio was 1.38 for the Org–MedDiet group versus 0.44 for the Conv–NoMedDiet group. •The average cost of the diet of Org–MedDiet participants was the highest. •The importance of promoting the MedDiet combined with organic food consumption is highlighted for individual health and environmental aspects but challenges regarding the cost remain.

*MedDiet, Mediterranean diet; DASH, Dietary Approach to Stop Hypertension; AHEI, Alternate Healthy Eating Index; DQI-I, Diet Quality Index International; Conv–NoMedDiet, Conventional consumers and non-MedDiet followers; Conv–MedDiet, Conventional consumers and MedDiet followers; Org–NoMedDiet, Organic consumers and non-MedDiet followers; Org–MedDiet, Organic consumers and MedDiet followers; GHGs, Greenhouse Gases; CF, Carbon Footprint; EF, Ecological Footprint; PANDiet, Probability of Adequate Nutrient intake; mPNNS-GS, modified Programme National Nutrition Santé-Guidelines Score*.

Most of the studies were from countries located in the Mediterranean basin; two studies were conducted in Italy (24, 25), two in Lebanon (27, 28), two in Spain (26, 29), one in France (30), one in Albania (22), and one was multicenter (the Netherlands, the United Kingdom, Germany, and Spain) (23). Five of the nine studies included in this analysis were cross-sectional observational studies (22, 25, 27, 28, 30), three were longitudinal observational studies (24, 26, 29), and one was experimental (clinical trial) (23). Most of the studies were conducted using the dietary consumption data from adults, and one study was conducted using the dietary consumption data from school children (24). Sample sizes vary from 289 to 22,866 subjects for the studies including adults and 172 subjects for the study including school children. The identified studies were conducted between 2017 and 2021.

#### Dietary Patterns

Two studies assessed the sustainability associated with the adherence to MedDiet (24, 27), one study assessed the sustainability of the adherence to MedDiet in combination with organic food consumption (30), one study assessed alterations in sustainability indicators resulting from an intervention promoting MedDiet (23), and the remaining studies compared MedDiet with other dietary patterns. The dietary patterns compared with MedDiet included the Dietary Approach to Stop Hypertension (DASH), the EAT-Lancet reference diet, the Nordic diet, the Western dietary pattern, the provegetarian dietary pattern, the high-protein dietary pattern, the dietary pattern based on the Alternate Healthy Eating Index (AHEI), and the Dietary Quality Index-International (DQI-I). The above-mentioned studies used dietary consumption data to calculate the adherence to each dietary pattern.

#### Sustainability Indicators

Most of the studies used life cycle assessments to obtain environmental sustainability indicators; therefore, reports of sustainability indicators related to the potential environmental impacts of food products were included in the dietary patterns during their entire life cycle (23–29). The environmental sustainability indicators included Greenhouse gases (GHGs) emissions (23, 25, 27–29), land use (23, 25, 29), energy use (23, 25, 27–29), water use (25, 27–29), carbon footprint (CF) (24), and ecological footprint (EF) (24). The CF is the amount of CO_2_ equivalent emissions (expressed in g CO_2_ eq) produced during the life cycle, and the EF is the area of land needed to regenerate the applied resources (expressed in m^2^). Four studies included indices obtained from environmental sustainability indicators as outcomes (23, 25, 26, 29). Grasso et al. (23) used the pReCiPe score, which is a weighted combination of GHGs emissions, land use, and fossil energy use. A sustainability score was applied by Gosso et al. (25) and Fresán et al. (29), and it was calculated by assigning 0 or 1 points to water, land, and energy use and GHGs emissions of each food product, using the sex-specific medians as the cut-offs (0 for upper values and 1 for lower ones). The sustainability score resulted from the sum of each component ranged from a total of 0 to 4 points, with higher scores indicating a less environmental impact. Fresán et al. (26) used a similar index, called the environmental footprints index, calculated in the same way as the sustainability score but in which participants were classified into quartiles, each of them ranking from 1 to 4. Similarly, the total environmental footprints index was created by summing the quartile values of all the four indicators (land, water, energy use, and GHGs emission); therefore, environmental footprints index ranked from 4 to 16 points.

Three studies included the cost of diet as an economical sustainability indicator (22, 26, 30). All the studies used the daily cost of diet as the main indicator; however, Seconda et al. (30) also reported the share of the budget allocated to foods by dividing the total cost of diet by the income reported by the participants.

Health-nutrition sustainability indicators were presented in two studies (26, 30). The rate of advancement period was used by Fresán et al. (26) as a heath indicator that measures the time by which a rate of a specific outcome is advanced or it is postponed among exposed subjects compared to unexposed individuals, conditional on being free from the outcome at the baseline. Nutrition indicators were used by Seconda et al. (30) to assess diet quality. Briefly, plant/animal protein ratio and three *a priori* dietary scores were computed: a diet quality index based on the Probability of Adequate Nutrient (PANDiet) intake that reflects the adequacy between nutrient intakes and French recommendations for 24 nutrients, the modified Programme National Nutrition Santé-Guidelines Score (mPNNS-GS) that reflects the level of adherence to the French food-based recommendation defined by the Programme National Nutrition Santé, and the dietary diversity score that evaluates the number of food groups consumed per day.

One index gathered the impact of the daily diet on health, environmental footprints index, and monetary costs; the overall sustainable diet index was designed and reported by Fresán et al. (26). Briefly, for the three aspects, a score from 0 to 3 points was given for each of them, the less suitable value for health, environment, and economy was given 0 points; 3 points for the healthiest daily diet, the one that produced less environmental footprints and the cheapest one. Proportional scores were given for the rest of the values. Summing those three values, the overall sustainable diet index was obtained ranging from 0 to 9 points, with 0 being the less suitable diet and 9 being the most appropriate diet.

#### Main Findings

The most consistent finding of the studies exploring sustainability based on dietary consumption data indicates that adherence to MedDiet is associated with higher environmental sustainability.

Naja et al. (27), Rosi et al. (24), and Fresán et al. (29) explored the association of the adherence to MedDiet with environmental sustainability indicators. In a sample of 2,610 adults from Lebanon, Naja et al. (27) found that higher adherence to MedDiet was associated with lower water use, lower GHGs emissions, and it was not associated with energy use. Fresán et al. (29) reported that higher adherence to MedDiet was associated with lower use of land, water, and energy, and reduced GHGs emissions. Surprisingly, Rosi et al. (24) found that higher adherence to MedDiet in a sample of 172 Italian school children was positively associated with CF and EF.

Grasso et al. (23) investigated whether food-related behavioral activation therapy applying MedDiet guidelines altered the food intake and the environmental impact of the diet in overweight adults with subsyndromal symptoms of depression. The intervention group altered food intake toward MedDiet; however, this effect resulted in no change in GHGs emissions, land use, and pReCiPe score, and a relative increase in the use of fossil energy.

Grosso et al. (25) studied the environmental impact of dietary patterns in an Italian cohort. The authors found that, except for DASH, the adherence to healthy dietary patterns (MedDiet and Nordic diet) and higher diet quality indices (AHEI and DQI-I) were associated with higher sustainability scores. They also found that higher adherence to MedDiet and AHEI was associated with lower GHGs emissions. Naja et al. (28) also found that adherence to MedDiet was associated with lower water use and GHGs emissions per 1,000 Kcal when compared to Western and high-protein dietary patterns. The environmental impact of the Western dietary pattern was also compared with MedDiet in a study from Fresán et al. (26), and it was shown that the Western dietary pattern was the most detrimental one for the environment, while the Provegetarian dietary pattern was the most beneficial one followed by the MedDiet.

Several of the studies also presented data on the contribution of food/food groups to the environmental sustainability indicators. Rosi et al. (24) showed that animal-based products represented 50% or more of the impact on the CF and EF. Similar results were observed by Grosso et al. (25) in which animal products represented more than half of the impact on GHG emissions, water use, and energy requirements. Naja et al. (28) reported that, within the MedDiet, whole dairy products had the highest percentage of contribution to water use, while vegetables contributed most to energy use and GHGs emissions. The authors explained these results by the relatively high consumption of vegetables within the Lebanese MedDiet and the fact that the production of vegetables requires more energy use and GHGs emissions than grains and fruits. In a later study (27), it was reported that red meat was the greatest contributor to water use, sugar-sweetened beverages were the main contributors to energy use, and red meat was the food group with the highest contributions to GHGs emissions.

Economic sustainability was assessed through the monetary cost. Llanaj et al. (22) analyzed the cost of the adherence to recommended dietary patterns and found that higher adherence to DASH, EAT-Lancet reference diet, or MedDiet was not associated with significant differences in cost. Fresán et al. (26), showed that MedDiet was the most expensive diet compared to the Western and Provegetarian dietary patterns. Seconda et al. (30) explored the cost of the adherence to MedDiet in combination with the consumption of organic food and observed that the average cost of consuming a MedDiet combined with organic food was the highest (MedDiet without organic food or no MedDiet compliance with or without organic food).

The health-nutrition pillar of sustainability was assessed by the study by Fresán et al. (26) and Seconda et al. (30). Fresán et al. (26) showed that the highest quartile of adherence to MedDiet exhibited the best rate advancement period (3.10 years gained), while the highest quartile of adherence to the Western dietary pattern showed the worst rate advancement period (1.33 years lost). Seconda et al. (30) demonstrated that the highest adherence to MedDiet (with or without combination with organic food) was associated with higher diet quality, adherence to recommendations, dietary diversity, and higher plant/animal protein ratio.

Fresán et al. (26) used an index that gathered the impact of all the analyzed aspects (health, environmental footprints, and monetary costs), the overall sustainable diet index. Using the overall sustainable diet index, the authors showed that MedDiet was the most sustainable option in comparison with Western and Provegetarian dietary patterns.

### MedDiet Sustainability Based on Dietary Scenarios

Out of the 28 articles included in this review, 11 analyzed MedDiet sustainability based on the models of dietary patterns or recommendations (dietary scenarios) (31–41). Relevant information from these articles is summarized in Table 3.

**Table 3:** Summary of studies reporting MedDiet sustainability using dietary scenarios.

**References**	**Location**	**Dietary scenarios**	**Sustainability indicators**	**Main findings**
Belgacem *et al*. (31)	Not applicable	•MedDiet •European dietary pattern •Western dietary pattern	•Land use •Water use •GHGs emissions •Eutrophication potential	•A shift from the European to the Mediterranean dietary pattern would lead to 10 m^2^/capita/day land savings, 240 L/capita/day water savings, 3 kg CO_2_/capita/day reduction in greenhouse gas emissions, and 20 g PO_4_eq/capita/day reductions in eutrophication potential. •A shift from the Western to the Mediterranean dietary pattern would lead to 18 m^2^/capita/day land savings, 100 L/capita/day water savings, 4 kg CO_2_/capita/day reduction in greenhouse gas emissions, and 16 g PO_4_eq/capita/day reduction in eutrophication potential.
Vanham *et al*. (32)	Nine Mediterranean countries (Spain, France, Italy, Greece, Turkey, Egypt, Tunisia, Algeria and Morocco)	•MedDiet •EAT-Lancet reference diet	•WF	•The EAT-Lancet diet requires less water resources than the MedDiet. In terms of water resources use, adherence to the former is thus more beneficial than adherence to the latter. •The EAT-Lancet diet reduces the current WF for all nations consistently, within the range−17–48%, whereas the MedDiet reduces the WF of the European countries, Turkey, Egypt and Morocco within the range of−4-−35%. •For the Maghreb countries Tunisia and Algeria, the Mediterranean diet WF is slightly higher compared to the current WF.
Gonzalez-García *et al*. (33)	Spain	•MedDiet •SEAD •NAOS	•CF •WF •Cost	•The dietary energy recommendation of the SEAD is greater than that of MedDiet and NAOS (11 and 15%, respectively), and SEAD also has greater animal source food content than the other two diets. •SEAD has a concomitantly higher CF, WF and cost scores in comparison with MD (+30, +23, and +21%, respectively) and NAOS (+15, +9, and +21%, respectively). •Adjusting recommendations to meet the suggested Spanish adult dietary energy of 2,228 kcal·capita^−1^·day^−1^ changed the environmental profiles of the diets, and the NAOS has the highest environmental impact. •Isocaloric diets had approximately the same cost. •Regardless of the dietary scenario, better scores were identified for the Spanish recommendations analyzed than those reported for other healthy diets identified in Europe.
Chapa *et al*. (34)	United States	•MedDiet •Healthy U.S. diet •Lacto-ovo vegetarian diet •Typical American diet	•NRF9.3 •NQI •FF •Global warming potential	•Vegetarian diets on average generated the lowest carbon footprint regardless of the NRF9.3, NQI and FF. •Animal products, including meat and dairy especially, and discretionary foods were identified as the specific food categories that contributed the most to the global warming potential.
Blackstone *et al*. (35)	United States	•MedDiet •Healthy US-style diet •Healthy vegetarian dietary pattern	•Global warming potential •Land use •Water use •Freshwater eutrophication •Marine eutrophication •Particulate matter or respiratory organics.	•The Healthy US-style dietary pattern and MedDiet pattern had similar impacts, except for freshwater eutrophication. •Freshwater eutrophication was 31% lower in the US pattern than the MedDiet pattern, primarily due to increased seafood in the MedDiet pattern. •All three patterns had similar water depletion impacts, with fruits and vegetables as major contributors. •For five of the six impacts, the Healthy vegetarian dietary pattern had 42–84% lower burdens than both the Healthy US-style dietary pattern and MedDiet pattern. •Reliance on plant-based protein and eggs in the Healthy vegetarian dietary pattern vs. emphasis on animal-based protein in the other patterns was a key driver of differences.
Ulaszewska *et al*. (36)	Italy	•MedDiet •New Nordic Diet	•GHGs emissions	•Consumption of high protein foods has a similar and comparable environmental impact to fruit and vegetable consumption. •Mediterranean Diet and New Nordic Diet had similar total values of GHG emissions.
van Dooren *et al*. (37)	Netherlands	•MedDiet •New Nordic Diet •Optimized Low Lands Diet	•GHGs emissions •Land use •Combined GHGE–LU Score •Health score	•An optimized Low Lands Diet has the same healthy nutritional characteristics (Health Score 123) as the Mediterranean Diet (122) and results in a lower environmental impact than the Mediterranean and New Nordic Diet (higher Combined GHGE-LU Score 121 vs. 90 and 91). •For optimized Low Lands Diet, GHGs emissions are 2.60 kg CO_2_eq per day and land use are 2.86 m^2^*year per day, which are the best scores of all diets analyzed.
•Vanham *et al*. (38)	•13 Mediterranean cities (Dubrovnik, Lyon, Athens, Jerusalem, Genova, Pisa, Bologna, Reggio Emilia, Ljubljana, Manresa, Zaragoza, Ankara and Istanbul)	•Healthy MedDiet •Healthy pesco-vegetarian MedDiet •Healthy vegetarian MedDiet	•WF	•Compared to reference situation, adoption of Healthy MedDiet (including meat), leads to WF reductions of −19–43%. The Healthy pesco-vegetarian MedDiet leads to WF reductions of −28–52%. The Healthy vegetarian MedDiet leads to WF reductions of −30–53%.
Blas et al. (39)	Spain and United States	•MedDiet •Typical American diet	•WF	•American diet has a 29% higher WF in comparison with the MedDiet, regardless of product's origin. •A shift to a Mediterranean diet would decrease the WF by 1,629 L/person/day in the US. A shift toward an American diet in Spain will increase the WF by 1,504 L/person/day.
Pairotti *et al*. (40)	Italy	•MedDiet •Italian average diet •Healthy consumption pattern •Vegetarian consumption pattern	•Cost •Energy use •CF	•When compared with the Italian average diet, the MedDiet revealed an improvement in environmental performance of 95.75 MJ (2.44%) and 27.46 kg CO_2_ equivalent (6.81%) per family. •The best overall environmental performance can be found with the vegetarian diet in which energy consumption is 3.14% lower and the carbon footprint 12.7% lower than the Italian average diet.
Rahmani *et al*. (41)	Iran	•Status-quo diet •MedDiet •WHO recommendations •WCRF recommendation	•Total changes in output •Environmental load	•Compared to Sattus-quo diet, total changes in output in WHO, WCRF and Mediterranean dietary scenarios were calculated to be 7010.1, 4802.8 and 3330.8 billion Rials respectively. •The environmental load increased for three dietary scenarios in comparison with the status-quo diet. The greatest and smallest environmental load occurred in WHO and Mediterranean dietary scenarios respectively.

*MedDiet, Mediterranean diet; SEAD, Southern European Atlantic diet; NAOS, Spanish dietary guidelines; WHO, World Health Organization; WCRF, World Cancer Research Fund; GHGs, Greenhouse Gases; WF, Water Footprint; CF, Carbon Footprint; NRF9.3, Nutrient Rich Foods Index 9.3; NQI, Nutritional Quality Index; FF, Fullness Factor™; GHGE-LU, Greenhouse Gases Emissions-Land Use*.

Studies were conducted using the recommendations or dietary patterns from countries located in the Mediterranean basin, Netherlands, and the United States; briefly, one study was conducted in the Netherlands (37), three studies in the United States (34, 35, 39), seven studies in the Mediterranean basin (32, 33, 36, 38–41), and one study with no specific location discernible (31). The identified studies were published between 2012 and 2021.

#### Dietary Patterns

Most of the studies compared the MedDiet scenario with other dietary patterns or recommendations, such as, the European dietary pattern (31), the Western dietary pattern (31), EAT-Lancet reference diet (32), the Southern European Atlantic Diet (SEAD) (33), the Spanish Dietary Guidelines (NAOS) (33), Healthy US diet (34, 35), Lacto-ovo vegetarian diet (34), typical American diet (34, 39), healthy vegetarian dietary pattern (35), New Nordic diet (36, 37), optimized Low Lands diet (37), Italian average diet (40), healthy consumption pattern (40), vegetarian consumption pattern (40), status-quo diet (Iran) (41), WHO recommended diet (41), and the diet recommended by World Cancer Research Fund (WCRF) (41). One study explored the sustainability of different MedDiet scenarios, such as Healthy MedDiet, healthy pesco-vegetarian MedDiet, and healthy vegetarian MedDiet (38).

#### Sustainability Indicators

Most of the studies reported environmental sustainability indicators, including land use (31, 37), water use (31, 35), GHGs emissions (31, 36, 37), eutrophication potential (31), water footprint (WF) (32, 33, 39), CF (33, 40), global warming potential (34, 35), freshwater eutrophication (35), marine eutrophication (35), particulate matter or respiratory organics (35), and energy use (40). The WF is an indicator of freshwater consumption (from rainfall, surface, and groundwater) that looks at direct and indirect water use of a producer or consumer and water resources appropriation (expressed in liters) (33). One study used a combined GHGs emissions-land use (GHGE-LU) score that was defined as the average of the GHGs emissions and LU score per diet (37, 43). One study reported the variation in environmental load (emission of GHGs, such as CO_2_, CH_4_, and N_2_O) expected in case of change for different dietary scenarios (41).

Sustainability was also assessed in the dimensions of economy and health nutrition. Economic sustainability was assessed in three studies, using the daily cost of diet (expressed in €·person^−1^·day^−1^ or €·family^−1^·month^−1^) (33, 40) or total changes in output (41). One study assessed the nutritional quality through the Nutrient Rich Foods Index 9.3 (NRF9.3) and Nutrient Quality Index (NQI), and satiety was assessed by the FullnessFactor™ (FF) (34). van Dooren et al. (37) used a health score to assess the healthiness of diets, based on the adequacy of the Dutch recommendations of ten nutritional indicators (food, nutrients, or energy).

#### Main Findings

Studies using dietary scenarios consistently found MedDiet as a sustainable pattern; although, it was not always considered superior to other healthy dietary patterns.

Vanham et al. (32) estimated the WF of MedDiet and EAT-Lancet reference diet in nine Mediterranean countries. The authors reported that the EAT-Lancet reference diet consistently reduces the current WF of the analyzed countries while MedDiet reduces WF to a smaller extent or even increases it. In a previous study, Vanham et al. (38) compared the WF of MedDiet scenarios with the reference situation in 13 Mediterranean cities and demonstrated that the adoption of MedDiet patterns (either including meat, pesco-vegetarian, or vegetarian) would reduce WF. Blas et al. (39) also compared the WF of MedDiet with the American diet and reported that the American diet has a 29% higher WF. The authors also reported that a shift to the Mediterranean diet would decrease the WF in the US, while a shift toward an American diet in Spain will increase the WF. Despite presenting a lower WF when compared to a typical American diet, the MedDiet presented a higher water depletion, and higher freshwater and marine eutrophication when compared with the Healthy US-style dietary pattern and the healthy vegetarian dietary pattern according to the study by Blackstone et al. (35). In this study (35), MedDiet presented a slightly lower global warming potential and land use, and slightly higher particulate matter than the Healthy US-style dietary pattern; however, MedDiet presented the worst environmental performance in all indicators when compared to healthy vegetarian dietary pattern. The authors mentioned that reliance on plant-based protein and eggs in the healthy vegetarian dietary pattern vs. emphasis on animal-based protein in the other patterns was a key driver of differences. A lacto-ovo vegetarian diet also performed better than other dietary patterns analyzed in the United States, including the MedDiet.

Chapa et al. (34) showed that Lacto-ovo vegetarian diet generated the lowest global warming potential regardless of the nutritional quality and satiety. Considering the nutritional quality and satiety, the authors concluded that high satiety foods can help prevent overconsumption and thus improve dietary CF. The authors also identified animal products, including meat and dairy, and discretionary foods as the specific food categories that contributed the most to the global warming potential. Similarly, Pairotti et al. (40) found that, when compared with the Italian average diet, the MedDiet revealed an improvement in energy use and in CF. Despite that, compared to the Italian average diet, the best overall environmental performance was found with the vegetarian diet in which energy use was 3.14% lower and the CF was 12.7% lower.

Gonzalez-García et al. (33) found that MedDiet had a lower CF and WF than SEAD and NAOS, the two recommended healthy dietary patterns in Spain. The SEAD presented the higher CF and WF explained by the greater animal source food content present in that dietary pattern. Belgacem et. al (31) compared three dietary scenarios and found that a shift from the European or Western dietary pattern to the MedDiet would lead to land and water savings, reduction in GHGs emissions, and eutrophication potential. Ulaszewska et al. (36) found comparable values of GHGs emissions in the MedDiet and the New Nordic diet. On the other hand, Rahmani et al. (41) observed that, in Iran, changing from the status-quo diet to MedDiet would increase the environmental load. Van Dooren et. al (37) noticed that an optimized low lands diet would result in a lower environmental impact (lower GHGs emissions, lower land use, and higher combined GHGE-LU score) with similar nutritional characteristics (measured by the health score) as the MedDiet.

Gonzalez-García et al. (33) analyzed the economic sustainability and, considering the isocaloric diets, the MedDiet, SEAD, and NAOS presented approximately the same cost. Pairotti et al. (40) indicated that MedDiet presented approximately the same cost as that of the Italian average diet.

### Mixed Studies

Out of the 28 articles included in this review, 4 analyzed MedDiet sustainability based on the models of dietary patterns or recommendations (dietary scenarios) in comparison with the national food consumption surveys (42–45). Relevant information from these articles is summarized in Table 4.

**Table 4:** Summary of studies reporting MedDiet scenario sustainability vs. other scenarios or dietary consumption.

**References**	**Participants; *n***	**Location**	**Dietary patterns**	**Sustainability indicators**	**Findings**
Blas et al. (42)	National representative sample; *n* = 8,000 households	Spain	•MedDiet •Spanish dietary pattern	•Multidimensional nutritional analysis •WF •Nutritional-Water productivity	•Spanish dietary pattern has 3 times more meat-dairy-sweet and 1/3 fewer fruits-vegetables than MedDiet. •Due to the high embedded water content in animal products, a shift toward a MedDiet would reduce the consumptive WF about 750 l/capita day. •MedDiet has better water-nutritional efficiency (NWP) than the current one: it provides more energy, fiber, and nutrients per liter of consumptive water.
van Dooren et al. (43)	National representative sample; (1–97 years); *n* = 5,958	Netherlands	•MedDiet •Dutch diet •Official “recommender” Dutch diet •Semi-vegetarian diet •Vegetarian diet •Vegan diet	•Health score •GHGs emissions •Land use •Combined GHGE–LU Score	•Consumption of meat, dairy products, extras, such as snacks, sweets, pastries, and beverages, are largely responsible for low Combined GHGE–LU Score and simultaneously, these food groups contribute to low health scores. •The Mediterranean diet is generally the health focus option with a high Combined GHGE–LU Score. •Health and Combined GHGE–LU Score of all six diets go largely hand in hand.
Germani et al. (44)	National representative sample; (0.1-97.7 years); *n* = 3,323	Italy	•MedDiet •INRAN-SCAI consumption	•CF •EF •WF •Cost	•MedDiet produce a lower environmental impact than the food consumption of the Italian population (CF, EF and WF). •The monthly expenditure of the MedDiet is slightly higher in the overall budget compared to the expenditure allocated to food by the Italian population.
Sáez-Almendros et al. (45)	National representative sample; *n* = 6,000 households	Spain	•MedDiet •Spanish dietary pattern •Western dietary pattern	•GHGs emissions •Land use •Energy use •Water use	•Increasing adherence to the MedDiet pattern in Spain will reduce GHGs emissions (72%), land use (58%) and energy consumption (52%), and to a lower extent water consumption (33%). •Adherence to a western dietary pattern implies an increase in all the descriptors between 12 and 72%.

*MedDiet, Mediterranean diet; INRAN-SCAI, Italian National Food Consumption Survey; WF, Water Footprint; NWP, Nutritional Water Productivity; GHGs, Greenhouse Gases; GHGE-LU, Greenhouse Gases Emissions-Land Use; CF, Carbon Footprint; EF, Ecological Footprint*.

Studies were conducted in countries located in the Mediterranean Basin and north of Europe; briefly, two studies were conducted in Spain, one study in Italy, and one study in the Netherlands. The identified studies were conducted between 2013 and 2019.

#### Dietary Patterns

All the studies compared MedDiet and other dietary patterns or recommendations with dietary consumption data obtained from national representative surveys. Apart from MedDiet, dietary scenarios explored in these studies included the Official “recommended” Dutch diet (43), the semi-vegetarian diet (43), the vegetarian diet (43), the vegan diet (43), and the Western dietary pattern (45). The dietary consumption patterns, obtained from the national representative samples, correspond to the Spanish dietary pattern (42, 45), the Dutch diet (43), and the real consumption of the Italian population (44).

#### Sustainability Indicators

Environmental sustainability indicators included WF (42, 44), GHGs emissions (43, 45), land use (43, 45), CF (44), EF (44), and WF (42, 44). One study used a combined GHGE-LU score (43).

Two studies included a health-nutrition indicator, the health score (43), and the multidimensional nutritional analysis (42). One study used an index that combines water use and nutritional values, the nutritional-water productivity (42). One study included the monetary cost (44).

#### Main Findings

MedDiet was consistently found to be a more sustainable option when a mixed approach, using dietary scenarios and data from food consumption surveys, was used.

Blas et al. (42) compared the WF of the Spanish dietary consumption with the MedDiet and demonstrated that a shift toward MedDiet would significantly reduce the WF. Furthermore, MedDiet presents better nutritional-water productivity than Spanish dietary consumption. The environmental sustainability of the Spanish dietary consumption was also compared with the sustainability of the adoption of a MedDiet pattern and a Western dietary pattern. Sáez-Almendros et al. (45) reported that increasing the adherence to the MedDiet pattern in Spain would reduce GHGs emissions, land use, energy consumption, and water consumption while increasing the adherence to a Western dietary pattern would increase all the descriptors.

van Dooren et al. (43) studied the environmental and health-nutrition sustainability of the Dutch diet and the other five dietary scenarios. Vegetarian diet and the vegan diet were the options with higher sustainability scores closely followed by MedDiet, which was the dietary pattern with the higher health score. MedDiet was considered, by the authors, the health focus option with a high GHGE-LU score.

When comparing the sustainability of the dietary consumption obtained through the Italian National Food Consumption Survey INRAN-SCAI 2005–06 with MedDiet recommendations, Germani et al. (44) showed that adherence to MedDiet may produce a lower environmental impact than the dietary consumption pattern of the Italian population. Despite the lower environmental impact, it was also shown that adherence to the MedDiet recommendations would result in a slightly higher cost when compared to the expenditure allocated to food by the Italian population, which may dampen the economic sustainability of MedDiet.

### Proposals of Methodological Approaches to Assess MedDiet Nutritional Sustainability

Out of the 28 studies identified through our strategy, four were proposals of methodological approaches to assess the MedDiet nutritional sustainability. Two studies were proposals of methodological approaches to assess the nutritional sustainability of the MedDiet (46, 47), and two studies were methodological proposals to assess the nutritional sustainability of MedDiet typical agro-food (48, 49). The identified proposals were published between 2013 and 2018. Relevant information is summarized in Tables 5, 6.

**Table 5:** Summary of proposed methodological approaches to assess MedDiet sustainability.

**References**	**Sustainability indicators**	
Donini et al. (46)	**Biochemical characteristics of food** •Vegetable/animal protein consumption ratios •Average dietary energy adequacy •Dietary Energy Density Score •Nutrient density of diet **Food Quality** •Fruit and vegetable consumption/intakes •Dietary Diversity Score	**Environment** •Food biodiversity composition and consumption •Rate of Local/regional foods and seasonality •Rate of eco-friendly food production and/or consumption **Lifestyle** •Physical activity/physical inactivity prevalence •Adherence to the Mediterranean dietary pattern **Clinical Aspects** •Diet-related morbidity/mortality statistics •Nutritional Anthropometry.
Dernini et al. (47)	**Nutrition and health** •Diet-related morbidity/mortality •Fruit and vegetable consumption/intake •Vegetable/animal protein consumption ratio •Dietary energy supply/intakes •Dietary diversity score •Dietary energy density score •Nutrient density/quality score •Food biodiversity composition and consumption •Nutritional anthropometry •Physical activity prevalence **Environment** •Water footprint •Carbon footprint •Nitrogen footprint •Biodiversity	**Economy** •Food consumer price index: cereals, fruit, vegetables, fish and meat •Cost of living index related to food expenditures: cereals, fruit, vegetables, fish and meat •Distribution of household expenditure per groups: food •Food self-sufficiency: cereals, fruit and vegetables •Intermediate consumption in the agricultural sector: nitrogen fertilizers •Food losses and waste **Society and culture** •Proportion of meals consumed outside home •Proportion of already prepared meals •Consumption of traditional products (e.g., proportion of product under PDO (Protected Designation of Origin) or similar recognized traditional foods) •Proportion of mass media initiatives dedicated to the knowledge of food background cultural value

**Table 6:** Summary of proposed methodological approaches to assess the sustainability of MedDiet's typical agro-food products.

**References**	**Sustainability indicators**	
Azzini et al. (48)	**Business distinctiveness of agro-food companies and food safety** •Distinctiveness for agro-food companies • Application of EU regulations, specific national laws, and voluntary requirements. •Primary production, marketing, and labeling • Nutritional macro and micronutrient content regulated by national and EU laws.	**Foodstuffs: the healthy-nutritional sustainability** •Nutritional sustainability index • Food specific nutritional indicators and their effects on health (Critical nutrients/“bioactive compounds,” whose concentrations are considered for calculating the macro-indicator on the nutritional quality for each group of foods. For details see original publication)
Capone et al. (49)	**Environmental criterion / indicators** •Land use and management • Application of soil conservation practices • Soil erosion protection •Input of nitrogen fertilizers • Input of plant protection products • Use of agricultural machinery •Biodiversity • Crop diversity • Number of farm animal species • Tree plant density • Herbaceous plant diversity • Presence of cover crops • Legume crop density • Patch average area • Semi-natural habitat surface • Duration of rotation • Diversity of varieties and animal breeds •Varietal diversity • Number of plant varieties threatened by genetic erosion • Number of animal races (varieties) • Number of animal races (varieties) threatened by genetic erosion •Energy • Energy intensity •Climate change • Final Energy consumption • Mineral fertilizers consumption • Pesticide consumption •Lubricant consumption • Plastic material consumption • Use of off-farm animal feeds •Use of chemical inputs • Nitrogen consumption • Use of total phosphorus pentoxide • Use of fungicides • Use of insecticides and acaricides • Use of herbicides •Environmentally sound management of production scraps, by-products, and waste • Method for management of production scraps, by-products, and waste **Economic criterion / indicators** •Income level and stability • Number of products and services produced by the farm • Distribution of the turnover among different products and services • Heterogeneity or affinity of products and services supplied • Index of commercial riskiness–suppliers • Index of commercial riskiness–customers	**Economic criterion / indicators (continued)** •Labor and employment • Index of localization •Investment • Specific investment for the improvement of sustainability performance •Profitability and productivity of production factors • Index of gross profitability per labor unit • Rate of return on invested capital • Enhancement rate • Rate of return of family labor **Socio-cultural criterion / indicators** •Life quality and human well-being of chain actors & corporate social and ethical responsibility • Companie's voluntary inclusion of social concerns in their activities •Women's participation in business production and management • Presence of women in business production and management •Social inclusion • Presence of disadvantaged groups in agribusiness •Relations with the local community • Collaboration with the local community, local authorities, and civil society • Social capital of agribusinesses •Promotion of local identity and transmission of traditional knowledge to the new generations • Activities other than agricultural production as a means for promoting the cultural identity • Preservation of traditions and local culture • Inter generation transmission of traditional knowledge •Workers' training planning throughout the chain • Workers' training throughout the chain •Implementation of training and foreign labor inclusion programs • Inclusion and training of foreign workers •Respect for animal welfare • Application of measures of animal welfare **Nutrition-health criterion / indicators** •Healthiness and food safety • Farm distinctiveness • Nutritional quality of solid agro-food material • Nutritional quality of liquid agro-food material • Nutritional quality by food groups (Critical nutrients, whose concentrations are considered for calculating the macro-indicator on the nutritional quality for each group of foods. For details see original publication)

#### Sustainability of Dietary Patterns

Dernini et al. (47) proposed a methodological approach to assess the sustainability of dietary patterns using MedDiet as a case study. The methodological approach was based on the results of the participatory process, conducted in 2011 and 2012 by the International Centre for Advanced Mediterranean Agronomic Studies-Mediterranean Agronomic Institute of Bari (CIHEAM MAI-Bari) and FAO in collaboration with the National Agency for New Technologies, Energy and Sustainable Economic Development, Italy (ENEA), Italian National Research Council (CNR), the National Institute for Research on Food and Nutrition, Italy (INRAN), the International Interuniversity Study Centre on Mediterranean Food Cultures (CIISCAM), Bioversity International, and World Wildlife Fund for Nature, Italy (WWF-Italy), in which the three dimensions of sustainability (economic, social, and environmental) were added to nutrition and health. Within these, four thematic areas were identified as sets of sustainability indicators. The list of sustainability indicators for each criterion that was established is reviewed in Table 5.

The sustainability indicators on the nutrition and health thematic area included diet-related morbidity/mortality, fruit and vegetable consumption/intake, vegetable/animal protein consumption ratio, dietary energy supply/intakes, dietary diversity score, dietary energy density score, nutrient density/quality score, food biodiversity composition and consumption, nutritional anthropometry, and physical activity prevalence. On the environment thematic area, the sustainability indicators aggregated WF, CF, nitrogen footprint, and biodiversity. The set of sustainability indicators on the economy thematic area were food consumer price index, cost of living index related to food expenditures, distribution of household expenditure per food group, food self-sufficiency, intermediate consumption in the agricultural sector (nitrogen fertilizers), and food losses and waste. Identified indicators in the thematic area of society and culture were the proportion of meals consumed outside the home, the proportion of already prepared meals, consumption of traditional products (e.g., the proportion of products under the protected designation of origin or similar recognized traditional foods), and proportion of mass media initiatives dedicated to the knowledge of food background cultural value.

Later, in 2016, Donini et al. (46), in the sequence of the above-mentioned work, identified, refined, and summarized some of the most relevant nutritional indicators to measure the sustainability of food consumption and dietary patterns using the MedDiet as a case of study. Five main thematic areas were identified and included biochemical characteristics of food, food quality, environment, lifestyle, and clinical aspects. Among those areas, 13 nutrition indicators of sustainability were identified and the definition, the methodology, the background, data sources, limitations, and references for each indicator were provided.

Sustainability indicators proposed for the “biochemical characteristics of food” thematic area were vegetable/animal protein consumption ratios, average dietary energy adequacy, dietary energy density score, and nutrient density of the diet. For the “food quality” thematic area, the indicators were fruit and vegetable consumption/intakes, and dietary diversity score. In the “environment” thematic area, the authors proposed as sustainability indicators the food biodiversity composition and consumption, rate of local/regional foods and seasonality, and rate of eco-friendly food production and/or consumption. Proposed indicators for “lifestyle” thematic area were physical activity/physical inactivity prevalence, and adherence to the Mediterranean dietary pattern; while for the “clinical aspects” of the nutritional sustainability, the authors proposed the diet-related morbidity/mortality statistics and nutritional anthropometry as indicators.

#### Nutritional Sustainability of MedDiet Typical Agro-Food Products

A methodological approach to assess the environmental, economic, socio-cultural, and health-nutrition sustainability of Apulian agro-food products was proposed by Capone et al. (49) in 2016.

Azzini et al., including the authors of the above-mentioned study, the latter published a study (48) on the health-nutrition dimension of the typical agro-food products. Two main aspects of health-nutrition sustainability were considered: (1) the business distinctiveness of agro-food companies and food safety and (2) the nutritional quality of foodstuffs. It is important to mention that this work seems to be a refinement of the indicators identified in the nutrition-health principle published in the work of Capone et al. (49).

The proposed indicators for health-nutrition sustainability are reviewed in Table 6. The business distinctiveness aspect refers to farms/companies (company-based approach). It includes indicators that are not specific to a single product and depend on the whole management of the agro-food company. To evaluate a company's distinctiveness and food safety, the application of different regulations and standards regarding food safety together with statutory, regulatory, and voluntary requirements, the origins of the raw materials used, and marketing and labeling were considered.

The second aspect, the nutritional quality, refers to each individual product (product-based approach). The nutritional quality of products was assessed taking into consideration their crucial nutrient content, these nutrients being specific for each food product category/group. The selection criteria for nutritional indicators in the nutritional quality aspect were based on secondary data from scientific literature and other relevant sources. The authors considered “bioactive compound” biomarkers, present in foodstuff, in relation to their effect on the health of individuals and groups.

## Discussion

This is the first scoping review of the methodological assessment of MedDiet nutritional sustainability. A previous study (18) systematically reviewed the studies on sustainable diets to identify the components of sustainability that were measured and the methods applied to do so. In this work, we reviewed the scientific literature to identify the main components of the nutritional sustainability of MedDiet and the methods that have been applied to assess those components. The concept of nutritional sustainability is broad and complex and encompasses the three dimensions of sustainability, environmental, economic, and socio-cultural, and also the health-nutrition dimension (8).

Through our search strategy, we identified 28 articles; 24 studies exploring the dimensions of nutritional sustainability of the MedDiet (22–45), and 4 proposing the methodological approaches to assess the nutritional sustainability of MedDiet (46, 47) or the sustainability of typical agro-foods of MedDiet (48, 49). From the 24 studies exploring the sustainability of MedDiet, none fully addressed the complexity of the four dimensions of nutritional sustainability (environmental, economic, socio-cultural, and health-nutrition). One of the methodological proposals to assess the nutritional sustainability of MedDiet (47) contemplated the four dimensions of nutritional sustainability, as well as one of the methodological proposals to assess the sustainability of typical agro-foods of MedDiet (49). Nevertheless, no study was identified, through our search strategy or through the list of citing articles, applying those methodological proposals. The remaining methodological proposals (46, 48) were further characterizations of the health-nutrition dimension of sustainability from the previously mentioned studies.

From the research articles, several sustainability indicators were identified. Most of the identified research articles reported sustainability indicators pertaining to the environmental dimension of nutritional sustainability (23–29, 31–45). Six studies (22, 26, 33, 40, 41, 44) reported economic sustainability indicators and six studies (26, 30, 34, 37, 42, 43) reported the sustainability indicators of the health-nutrition dimension of nutritional sustainability. Two studies used indices that combined indicators from the environmental and health-nutrition components of sustainability (26, 42). No studies have reported indicators regarding the socio-cultural dimension. These results are not surprising, due to the large attention that the environmental dimension of sustainability has received over time and are in line with the results obtained in the systematic review of Jones et al. (18) where environmental indicators were reported in most of the identified studies; substantial less studies reported economic sustainability indicators and indicators of the socio-cultural dimension, such as the examination of cultural heritage and skills, equity, and rights, were almost entirely lacking.

Two of the leading threats to global health are climate change and non-communicable diseases, both of which are inextricably linked to diet (20, 50); in this sense, nutritional sustainability goes along with the One Health concept where human, animal, and the environmental health are intimately linked (51). The One Health approach, by definition, encompasses many fields, including, but not limited to, health, ecology, agriculture and sustainability, economics, anthropology, and the social sciences (52). All those disciplines are also included in the assessment of nutritional sustainability. Assessing the environmental dimension of sustainability is of utmost importance. The emissions of the global food system (from food production to consumption) are estimated to account for 21–37% of total human-induced GHGs emissions, 70% of freshwater use, increased eutrophication, and consumption of 35% of ice-free land, and it is also the greatest cause of deforestation and biodiversity loss, thereby contributing to the detrimental effects on natural resources (19, 24, 53). Recently, the report of the EAT-Lancet Commission on healthy diets from sustainable food systems (1) indicated that food systems are the major driver of environmental degradation and further food production should use no additional land, safeguard existing biodiversity, reduce consumptive water use and manage water responsibly, substantially reduce nitrogen and phosphorus pollution, produce zero carbon dioxide emissions, and cause no further increase in methane and nitrous oxide emissions. Sustainability indicators to assess those recommendations were found in the articles included in this review. Among the indicators cited, the most used were related to global warming potential (GHGs emissions and CF) (23–29, 31, 33, 35–37, 40, 41, 43–45), followed by water (25–29, 31–33, 35, 38, 39, 42, 44, 45), land (23, 25, 26, 29, 31, 35, 37, 43, 45), and energy use (23, 25–29, 40, 45). Our findings are in line with the previous studies where the global warming potential of diets was by far the most commonly measured environmental sustainability indicator, with land, energy, and water use also frequently assessed (18). Considering the detrimental impacts that food systems have on the environment, it is not surprising to observe the abundance of those sustainability indicators in the identified literature. Most of the studies used the life cycle assessment (LCA) approach to obtain environmental sustainability indicators. This finding is consistent with the literature on the subject, where LCA is the most commonly used approach (18–20, 54). Despite being the most commonly used approach, LCA methodology is not free from limitations (55), and other methodologies to assess sustainability, such as the modeling approaches, integrated analytical frameworks, and the proposed adaptive, participatory methods, have been proposed (18).

From the environmental perspective, many of the identified studies consistently found that MedDiet is a sustainable option (25–31, 33, 38–40, 42–45). Nevertheless, some studies relying on dietary consumption data or dietary scenarios reported that in some cases, other dietary patterns had a similar or better environmental performance (22, 25, 26, 28, 32, 34–37, 41), while the mixed studies, based on dietary consumption and dietary scenarios, indicated MedDiet as the most environmentally friendly option (42–45). Studies examining the impact of foods on environmental sustainability reported animal food sources as the food category with the most deleterious environmental effects (25, 34, 35). As previously mentioned, MedDiet is a dietary pattern characterized by moderate consumption of eggs, poultry, and dairy products (cheese and yogurt) and low consumption of red meat (13, 14). Furthermore, in its present update, the MedDiet pyramid reflected multiple environmental concerns and strongly emphasizes a lower consumption of red meat and bovine dairy products (13, 56).

Six studies (22, 26, 33, 40, 41, 44) measured the cost associated with the adherence to MedDiet as a measure of economic sustainability. Those studies shed some light on the economic tradeoffs of adhering to MedDiet. In two of the studies (26, 44), adherence to the MedDiet, compared to other patterns of dietary consumption, was associated with a higher cost; yet, in one study (33), it was proposed that isocaloric diets have approximately the same cost. These results may be explained by the different methodological approaches used in each study but are most likely explained by the dietary patterns compared to the MedDiet. The MedDiet was more expensive than the Western dietary pattern and the Provegetarian dietary pattern (26), slightly more expensive than the dietary consumption of the Italian population (44); no significant differences were observed between the MedDiet, the SEAD, and the NAOS (33). Monetary cost is one of the key factors in food choice and it is the main factor in shaping the consumer demand; therefore, it will affect consumer preferences and options for a sustainable dietary pattern (18, 57). Food prices condition the affordability of sustainable diets. Low prices reduce the income of producers, reduce their ability to invest, and may hinder the development of a sustainable food system. From the sustainability point of view, price is ambivalent; therefore, it is important to guarantee the accessibility and affordability to food choices in order to ensure economic sustainability but at the same time, the affordability may have negative environmental impacts by not discouraging food waste (58). In line with our findings, there is evidence indicating that MedDiet is not necessarily associated with higher overall dietary costs (59).

The health-nutrition dimension of nutritional sustainability of MedDiet was assessed in six studies (26, 30, 34, 37, 42, 43). Fresán et al. (26) used the advantage of a longitudinal study to explore the time by which a rate of an outcome (death, non-fatal cardio vascular disease (CVD) myocardial infarction or stroke, non-fatal breast cancer, or type 2 diabetes mellitus, whichever occurred first) is advanced or is postponed within individuals exposed to different dietary patterns. The NRF9.3 and NQI were also used to assess the diet quality in combination with FF, to quantify the satiety response of food (34). van Dooren et al. (37, 43) used a health score, that was composed by the ratio between the consumption and the recommendations for some food groups, nutrients, and energy. Regardless of the methodological differences, MedDiet was associated with a better performance in the health-nutrition dimension. MedDiet has been consistently shown to be a healthy dietary pattern that may reduce risk related to non-communicable diseases (60); and therefore, adherence to the MedDiet or other healthy dietary patterns may be associated with the sustainability of healthcare systems.

The absence of exploration regarding the socio-cultural dimension of sustainability in the identified literature is particularly important, given the critical role of society and culture in the MedDiet. The relevance of this dimension is so clear that MedDiet was acknowledged by UNESCO as an intangible cultural heritage (61). According to UNESCO, MedDiet is a way of life that encompasses a set of skills, knowledge, rituals, symbols, and traditions, ranging from landscape to the table. Eating together is the foundation of the cultural identity and continuity of communities throughout the Mediterranean Basin. The MedDiet emphasizes values of hospitality, neighborliness, intercultural dialogue and creativity, and a way of life guided by respect for diversity (17). Despite its increasing popularity worldwide, adherence to the MedDiet is decreasing due to multifactorial influences, such as globalization, population growth, and socio-economic changes. Food chain modernization has increased productivity and resulted in a substantial transformation of lifestyles as a consequence of rising incomes, urbanization, and changes in the agricultural and food sectors. Those changes threaten seriously the transmission and preservation of the MedDiet heritage to present and future generations (17). Measuring the sustainability of the socio-cultural dimension is paramount for the preservation of MedDiet.

Six studies (23, 25, 26, 37, 42, 43, 51) combined indicators to provide a “sustainability index.” Most of the studies combined environmental indicators into environmental sustainability indices (23, 25, 37, 43). Fresán et al. (26) designed an index that gathered the impact of the daily diet on the analyzed aspects: health, environmental footprints, and monetary costs. Blas et al. (42) proposed the nutritional water productivity (NWP) that links water and nutrition. The development of indices that combine all the dimensions of nutritional sustainability may facilitate its assessment and the comparability of different dietary patterns or food products.

We did not identify studies that used methodological approaches covering all the conceptual framework of nutritional sustainability of MedDiet; instead, we identified studies that assessed some dimensions of MedDiet nutritional sustainability. Heterogeneity in the indicators used was found, particularly in the environmental dimension. Studies on the economic and health-nutrition dimensions are less frequent and absent in the socio-cultural dimension. Our findings call for the development of harmonized methodologies for the assessment of MedDiet nutritional sustainability. Indeed, the methodological approach proposed by Dernini et al. (47) identified indicators to assess the sustainability of the four dimensions that should be considered. Despite being comprehensive and complete, no indication is given regarding the weight of each dimension or the indicator for a sustainability score; although the authors mention that the methodological approach requires to be tested and further refined in a group of selected Mediterranean countries, indicating that this is an ongoing work.

Traditional and typical agro-food products are at the core of MedDiet (49). A typical agro-food product is characterized by historical and cultural features and by physical attributes that are deep-rooted to the territory of origin encompassing much more than organoleptic qualities. In the last years, we have observed a deep transformation in consumer perception and in the demand for typical agro-food products. The retrieval of typical and traditional foods represents an attempt to recover the safety and social aspects of eating habits. To form positive attitudes and expectations toward food, consumers need to be assured and informed about the production and transformation processes as well as about their origin and the symbolic values they encompass (62). Typical agro-food products contribute directly and indirectly to the sustainability of the MedDiet in the Mediterranean basin (49). Considering those aspects, we identified two works related to the sustainability of typical agro-food products (48, 49). Capone et al. (49) proposed a comprehensive approach to assess the sustainability of typical agro-food products of the MedDiet. This methodological proposal englobes all the dimensions of sustainability that are explored in our study. The identified work of Azzini et al. (48) seems to provide clarification to the health-nutrition dimension mentioned in the work of Capone et al. (49).

In this work, sustainability was assessed in the environmental, economic, sociocultural, and health-nutrition dimensions. Considering the included literature, environmental sustainability was assessed and defined as the ability to use fewer resources (23, 25–29, 31–33, 35, 37–40, 42–45) to produce less byproducts (23–29, 31, 33–37, 40, 41, 43–45). Economic sustainability was defined as the ability to promote economic growth (41) or the accessibility to the consumers (22, 26, 33, 40, 44). The Heath-nutrition dimension was defined as the capability to provide adequate nutrition (30, 37, 42, 43), promote health, and prevent disease (26). Despite not being assessed, the socio-cultural dimension of sustainability encompasses historical remains and values, local culture, and traditions; therefore, it was defined as the ability to preserve them (63). Nutritional sustainability is an umbrella term that can take several meanings depending on the dimension that is assessed.

Several considerations must be made regarding the findings of this study. Most of the studies identified are from the countries located in the Mediterranean basin and the remaining are from Northern Europe and the United States. While it is not surprising to find studies regarding MedDiet sustainability in the countries of its origin, MedDiet is recommended worldwide as a sustainable dietary option (64); therefore, studies on other regions are needed. Comparisons are difficult due to the heterogeneity of the indicators used in the identified studies and no studies used a comprehensive approach that explores nutritional sustainability in all dimensions. Harmonization is essential for the comparison of results; yet, a significant degree of flexibility is also needed to allow for the wide application of an instrument to assess the nutritional sustainability of diets or food products that are, by nature, dynamic. Identified studies did not provide examples of approaches to combine all the indicators of sustainability. Identified articles were published between 2012 and 2021, highlighting the recent interest in the subject. Despite a significant body of literature that meets the inclusion criteria for this review, more work is needed to establish a consensual approach to assess the nutritional sustainability of MedDiet and to compare it with other dietary patterns.

Our scoping review has some limitations. A search was performed only in two electronic databases (Scopus and PubMed); therefore, relevant works may have been missed. Gray literature could be an informative source of evidence to this study; however, the sizable amount of gray literature in the field could have dumped the feasibility of the work. The search strategy was broad enough to capture a significant body of literature in the area, yet it is possible that studies assessing the sustainability indicators but not mentioning the word sustainability (or related words) have not been captured.

Our study reviewed for the first time the assessment of the nutritional sustainability of MedDiet. From a general perspective, there is sufficient evidence to state that MedDiet is a nutritional sustainable option. Methodological assessment of nutritional sustainability is challenging and involves multidisciplinary approaches. To the best of our knowledge, no research has been made assessing MedDiet in all the dimensions of the complex concept, that is nutritional sustainability. In its concept, nutritional sustainability is differentiated from other concepts combining nutrition and sustainability; it does not contradict with other similar concepts (sustainable diet and sustainable food systems) but aggregates concepts from them. MedDiet nutritional sustainability needs to attract sufficient political attention and become a core priority in the shaping of agriculture, food, and nutrition policies; for that, research needs, in a comprehensive way, to reflect the complexity of the nutritional sustainability concept. Integrating health and nutrition, environmental, economic, and socio-cultural considerations across scales and contexts can offer a more complete understanding of the opportunities and barriers to achieving nutritional sustainability not only in MedDiet but also in other dietary patterns and food products.

## Data Availability Statement

The original contributions presented in the study are included in the article/Supplementary Material, further inquiries can be directed to the corresponding authors.

## Author Contributions

CP-N wrote the first draft of the manuscript. The data acquisition of the article and analysis of its content has been made by a consensus between CP-N and CG. CS and CG conceived and designed the study. All the authors had revised the manuscript.

## Funding

This work was supported by the AgriFood XXI project (NORTE-01-0145-FEDER-000041) co-financed by the European Regional Development Fund through NORTE 2020 and by the project UIDB/CVT/00772/2020 funded by the Fundação para a Ciência e Tecnologia (FCT). The CECAV is supported by FCT/UIDB/CVT/00772/2020. The CIAFEL is supported by FCT/UIDB/00617/2020. The CITAB is supported by FCT/UIDB/04033/2020. The CQ-VR is supported by FCT UIDB/00616/2020 and UIDP/00616/2020. The CP-N is supported by an AgriFood XXI project post-doctoral fellowship.

## Conflict of Interest

The authors declare that the research was conducted in the absence of any commercial or financial relationships that could be construed as a potential conflict of interest.

## Publisher's Note

All claims expressed in this article are solely those of the authors and do not necessarily represent those of their affiliated organizations, or those of the publisher, the editors and the reviewers. Any product that may be evaluated in this article, or claim that may be made by its manufacturer, is not guaranteed or endorsed by the publisher.
